# Effects of TENS with home exercise improve pain and muscle strength in older adults with pre-radiographic to mild knee osteoarthritis

**DOI:** 10.1038/s41598-025-23670-z

**Published:** 2025-11-14

**Authors:** Natsuki Katagiri, Kotaro Kawashima, Mizuki Hamasuna, Minoru Motoyama, Tomofumi Yamaguchi

**Affiliations:** 1 Department of Rehabilitation Medicine, Juntendo University Graduate School of Medicine, Tokyo, Japan; 2 Japan Society for the Promotion of Science, Tokyo, Japan; 3 Department of Physical Therapy, Juntendo University Graduate School of Health Science, Tokyo, Japan; 4 Department of Physical Therapy, Human Health Sciences, Graduate School of Medicine, Kyoto University, 53 Shogoin-kawahara-cho, Sakyo-ku, Kyoto, 606-8507 Japan; 5 Department of Physical Therapy, Juntendo University Faculty of Health Science, Tokyo, Japan

**Keywords:** Knee osteoarthritis, Pain management, Randomized controlled trial, Rehabilitation, Transcutaneous electrical nerve stimulation, Geriatrics, Musculoskeletal system

## Abstract

To date, no large-scale studies have been conducted on pre-radiographic to mild cases of knee osteoarthritis. Therefore, this randomized, double-masked, sham-controlled trial investigated the effects of transcutaneous electrical nerve stimulation (TENS) combined with home exercise in older individuals with pre-radiographic to mild knee osteoarthritis (Kellgren–Lawrence grade ≤ 2). A total of 126 participants experiencing knee pain were randomly assigned to the TENS (*n* = 64) or sham TENS group (*n* = 62), both combined with home exercise. The main outcome was knee pain during activity, measured via a visual analog scale (VAS). Both groups showed significantly decreased VAS scores during activity, with greater reductions in the TENS group than in the sham TENS group. Compared with the sham TENS group, the TENS group also showed significantly increased knee extensor strength in the painful knee, with no changes in the non-painful knee. No significant changes were observed in gait ability, physical activities, activities of daily living, and mental health between the groups. In conclusion, TENS combined with home exercise can reduce knee pain and increase knee extensor strength in older individuals with pre-radiographic to mild knee osteoarthritis.

## Introduction

Osteoarthritis affects more than 500 million people worldwide, with over 260 million having knee osteoarthritis^1,2^. The primary symptom is chronic pain, and effects extend beyond the knee joint. Chronic pain can adversely affect overall health, including social functioning, energy levels, and vitality, which negatively affect quality of life^3,4^. Therefore, pain management for knee osteoarthritis is necessary to improve an individual’s quality of life and mitigate the broader health implications associated with the condition.

The goal of pain management in osteoarthritis should be to reduce symptoms, improve function, and prevent long-term disability. However, current pain management strategies typically target patients with late-stage osteoarthritis^2^. Advanced stages of osteoarthritis are associated with bone deformity and a lower potential for pain improvement^5^. Therefore, to effectively manage early symptoms of osteoarthritis, it is crucial to intervene from the pre-radiographic stage before patients visit medical facilities for rehabilitation.

Guidelines for managing knee osteoarthritis recommend non-pharmacological interventions, such as exercise therapy^6^. Alongside therapeutic exercise, other established non-pharmacological interventions include patient education aimed at improving self-management^7^, manual therapy to enhance joint mobility^8^, and the use of knee braces for mechanical support and pain relief^9^. Home exercise is cost-effective and requires no special equipment; additionally, it is particularly effective for managing symptoms of knee osteoarthritis in community-dwelling older adults^6,10–12^. However, adherence to and effectiveness of exercise therapy can be limited by exercise-induced pain. We hypothesized that by providing analgesia, transcutaneous electrical nerve stimulation (TENS) could serve as an adjuvant therapy to enhance participation in and benefits of the home exercise program.

Electrical stimulation is a widely used therapeutic approach that modulates neuromuscular activity and promotes functional recovery in clinical populations^13,14^. One such modality, TENS, is a widely used conservative intervention that effectively reduces pain and improves knee function and mobility in patients with knee osteoarthritis^15–19^. However, recent large-scale multicenter randomized controlled trials have examined the effectiveness of TENS in patients with Kellgren–Lawrence grade ≥ 2, finding no significant differences compared to results of the control group^20^. Moreover, previous research has highlighted that structural limitations in advanced osteoarthritis are a key factor underlying the lack of TENS efficacy^21^. This could be due to the diversification of pain mechanisms with osteoarthritis progression—specifically, the addition of central sensitization to nociceptive pathways^22^—which may reduce TENS efficacy.

These findings highlight the importance of early intervention and support tailoring treatment to each patient’s symptom stage. Furthermore, they indicate that TENS alone may be insufficient for pain relief in patients with more advanced symptoms. Therefore, our study was designed to test the hypothesis that TENS could serve as an effective adjuvant therapy: by providing analgesia, it may reduce movement-evoked pain, enabling patients to engage more effectively in therapeutic exercises and enhancing the overall benefits of the program. To the best of our knowledge, no large-scale studies have yet investigated pre-radiographic to mild cases of knee osteoarthritis, classified as Kellgren–Lawrence grades 1 and 2.

Therefore, this study aimed to evaluate the effects of TENS combined with home exercise therapy on knee pain during activity, as well as on secondary outcomes including muscle strength, daily physical activity, pain-related interference with daily living, and mental health in individuals with pre-radiographic to mild osteoarthritis who were not undergoing rehabilitation. We also hypothesized that, in individuals with pre-radiographic to mild knee osteoarthritis, adding TENS to exercise therapy would yield greater improvements in knee pain and extensor strength than exercise alone, primarily by reducing pain during training sessions. We further theorized that these primary effects would lead to secondary improvements in physical activity, mental health, and participation in activities of daily living.

## Results

### Baseline participant characteristics

**Fig. 1:**
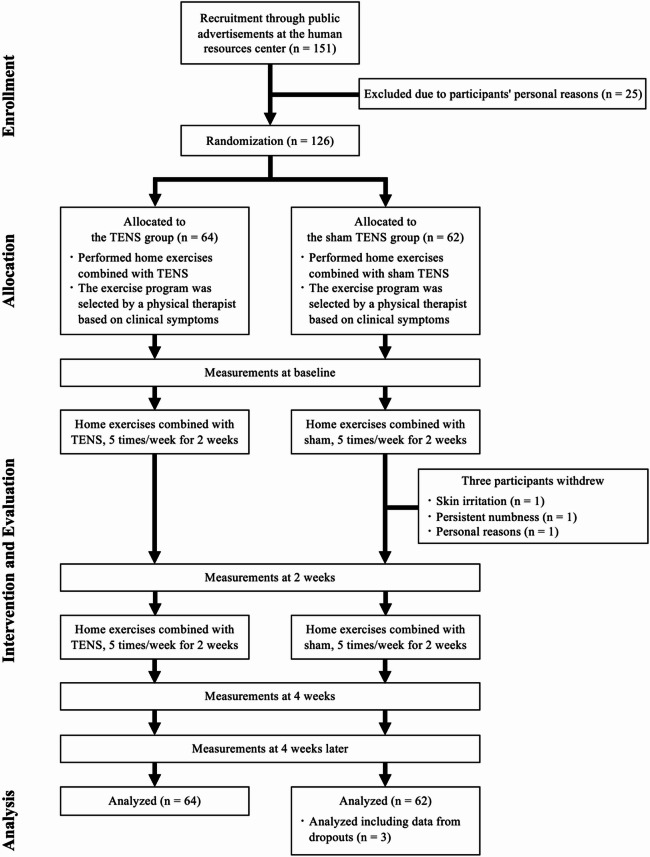
Study design and flowchart of participant inclusion in the trial. TENS, transcutaneous electrical nerve stimulation.

A total of 151 eligible participants were included in this study. Of these, 25 (16.6%) were excluded for personal reasons before group assignment. Therefore, 126 participants (TENS group, *n* = 64; sham TENS group, *n* = 62) were included in the analysis. Three participants withdrew during the intervention because of skin irritation, persistent numbness, and personal reasons (Fig. 1); however, the analysis was conducted using mixed models, which accounted for missing data. Of the participants, two experienced adverse events: one had skin irritation, and the other reported persistent numbness.

Adherence to the intervention was analyzed in 121 participants, after excluding two with missing questionnaire data. Within this cohort, 119 participants (98.3%) completed all 20 prescribed sessions, whereas the remaining two participants, one from each group, completed 19 of 20 sessions.

The correct group guess rates for the TENS and sham TENS groups (*n* = 62 and 57, respectively) were 58.1% (36 participants) and 38.6% (22 participants), respectively. Participant blinding was deemed effective, given the lack of a statistically significant association between actual and guessed group allocations (χ^2^_(1)_ = 0.138, *P* = 0.711).

Table 1 shows the mean baseline characteristics of the participants. Tables 2 and 3 present the primary and secondary outcomes, along with the results for each group and the estimated effect sizes with their 95% confidence intervals.

**Table 1:** Baseline participant characteristics continuous data are expressed as mean (SD, standard deviation).

Characteristic	TENS group (*n* = 64)	Sham TENS group (*n* = 62)
Age, y (SD)	69.72 (4.18)	70.66 (3.44)
Women, n (%)	26 (40.63)	30 (48.39)
Height, cm (SD)	165.75 (9.00)	163.11 (7.21)
Weight, kg (SD)	65.02 (12.83)	61.21 (10.38)
BMI, kg/m^2^ (SD)	23.50 (3.25)	22.93 (3.06)
K/L grade 1, n (%)	26 (40.63)	25 (40.32)
K/L grade 2, n (%)	38 (59.38)	37 (59.68)

Categorical data are expressed as n (%).

TENS, transcutaneous electrical nerve stimulation; BMI, body mass index; K/L, Kellgren–Lawrence.

**Table 2:** Means, standard deviations, mean differences, confidence intervals, and effect sizes of the variables related to the primary and secondary outcomes at all time points.

Measure	Testing time	TENS group			Sham TENS group		
		Mean (SD)	MD (95% CI)	ES (d)	Mean (SD)	MD (95% CI)	ES (d)
VAS score (cm)	Baseline	5.67 (1.61)	n/a	n/a	5.24 (1.83)	n/a	n/a
	2 weeks	3.66 (1.53)	2.02 (1.46 to 2.57)*	1.28	4.30 (2.36)	0.94 (0.17 to 1.71)*	0.45
	4 weeks	2.84 (1.28)	2.83 (2.27 to 3.38)*	1.95	3.91 (2.31)	1.33 (0.56 to 2.10)*	0.64
	4 weeks later	2.90 (1.43)	2.77 (2.21 to 3.33)*	1.82	3.35 (2.39)	1.89 (1.12 to 2.66)*	0.89
Painful knee extensor strength (N)	Baseline	235.56 (88.17)	n/a	n/a	241.75 (102.81)	n/a	n/a
	2 weeks	253.13 (95.30)	− 17.57 (− 40.64 to 5.51)	− 0.19	241.98 (89.81)	− 0.23 (− 24.53 to 24.08)	0.00
	4 weeks	294.92 (102.01)	− 59.35 (− 82.43 to − 36.28)*	− 0.62	252.68 (99.90)	− 10.92 (− 35.08 to 13.24)	− 0.11
	4 weeks later	273.02 (102.40)	− 37.46 (− 60.53 to − 14.38)*	− 0.39	246.19 (97.15)	− 4.44 (− 28.74 to 19.87)	− 0.04
Non-painful knee extensor strength (N)	Baseline	288.13 (109.35)	n/a	n/a	269.12 (111.99)	n/a	n/a
	2 weeks	269.65 (87.10)	18.48 (− 25.83 to 62.80)	0.18	246.42 (104.79)	22.70 (− 29.58 to 74.98)	0.21
	4 weeks	290.95 (94.04)	− 2.83 (− 47.14 to 41.49)	− 0.03	275.16 (127.23)	− 6.04 (− 58.95 to 46.87)	− 0.05
	4 weeks later	281.06 (93.62)	7.07 (− 37.25 to 51.38)	0.07	261.82 (117.35)	7.30 (− 45.19 to 59.78)	0.06
Gait speed (m/sec)	Baseline	1.86 (0.23)	n/a	n/a	1.87 (0.40)	n/a	n/a
	2 weeks	1.88 (0.41)	− 0.02 (− 0.15 to 0.11)	− 0.06	1.86 (0.33)	0.01 (− 0.10 to 0.11)	0.03
	4 weeks	1.93 (0.20)	− 0.07 (− 0.20 to 0.06)	− 0.32	1.85 (0.51)	0.02 (− 0.09 to 0.12)	0.04
	4 weeks later	1.90 (0.24)	− 0.03 (− 0.16 to 0.09)	− 0.17	1.90 (0.40)	− 0.03 (− 0.14 to 0.08)	− 0.07
TUG test time (sec)	Baseline	6.66 (1.26)	n/a	n/a	7.13 (1.82)	n/a	n/a
	2 weeks	6.60 (1.61)	0.07 (− 0.56 to 0.69)	0.04	6.72 (1.49)	0.41 (− 0.21 to 1.03)	0.25
	4 weeks	6.39 (1.28)	0.28 (− 0.35 to 0.90)	0.21	7.14 (2.25)	− 0.01 (− 0.64 to 0.62)	0.00
	4 weeks later	6.97 (1.31)	− 0.30 (− 0.93 to 0.32)	− 0.24	6.76 (1.59)	0.37 (− 0.26 to 0.99)	0.22
PDAS score (arb.)	Baseline	11.80 (11.47)	n/a	n/a	11.07 (9.10)	n/a	n/a
	2 weeks	8.26 (7.11)	3.54 (1.20 to 5.89)*	0.35	9.17 (6.86)	1.89 (− 0.53 to 4.32)	0.23
	4 weeks	8.15 (6.84)	3.64 (1.31 to 5.92)*	0.37	9.31 (6.91)	1.76 (− 0.68 to 4.12)	0.21
	4 weeks later	7.56 (7.10)	4.23 (1.91 to 6.56)*	0.42	8.73 (7.93)	2.33 (− 0.09 to 4.76)	0.27
HADS-A score (arb.)	Baseline	3.77 (3.36)	n/a	n/a	4.81 (3.02)	n/a	n/a
	2 weeks	5.41 (3.19)	− 1.64 (− 3.06 to − 0.22)*	− 0.50	4.01 (2.70)	0.80 (− 0.52 to 2.11)	0.28
	4 weeks	2.73 (2.19)	1.03 (− 0.39 to 2.45)	0.37	4.57 (3.09)	0.24 (− 1.09 to 1.57)	0.08
	4 weeks later	3.52 (2.65)	0.25 (− 1.17 to 1.67)	0.08	3.86 (2.62)	0.95 ( − 0.37 to 2.27)	0.34
HADS-D score (arb.)	Baseline	5.19 (3.79)	n/a	n/a	5.80 (3.39)	n/a	n/a
	2 weeks	6.91 (3.71)	− 1.72 (− 3.25 to − 0.18)*	− 0.46	5.75 (2.79)	0.04 (− 1.45 to 1.54)	0.02
	4 weeks	4.42 (2.78)	0.77 (− 0.77 to 2.30)	0.23	5.78 (3.18)	0.02 (− 1.49 to 1.53)	0.01
	4 weeks later	3.86 (2.44)	1.33 (− 0.21 to 2.87)	0.42	5.94 (3.05)	− 0.14 (− 1.64 to 1.35)	− 0.04
PCS score (arb.)	Baseline	16.06 (10.87)	n/a	n/a	18.80 (9.66)	n/a	n/a
	2 weeks	22.48 (9.29)	− 6.42 (− 10.69 to − 2.15)*	− 0.63	17.92 (8.77)	0.89 (− 2.84 to 4.61)	0.1
	4 weeks	14.70 (8.53)	1.36 (− 2.91 to 5.63)	0.14	19.43 (10.25)	− 0.63 (− 4.41 to 3.16)	− 0.06
	4 weeks later	17.44 (8.83)	− 1.38 (− 5.64 to 2.89)	− 0.14	16.16 (9.38)	2.65 (− 1.10 to 6.39)	0.28

SD, standard deviation; MD, mean difference (baseline minus each time point); CI, confidence interval; ES, effect size; d, Cohen d; VAS, visual analog scale; TUG, timed up-and-go; PDAS, pain disability assessment scale; HADS-A, hospital anxiety and depression scale-anxiety; HADS-D, hospital anxiety and depression scale-depression; PCS, pain catastrophizing scale; arb., arbitrary units.

*Statistically significant difference, *P* < 0.05.

**Table 3:** Means, standard deviations, mean differences, confidence intervals, and effect sizes of the variables related to physical activity.

Measure	Testing time	TENS group			Sham TENS group		
		Mean (SD)	MD (95% CI)	ES (d)	Mean (SD)	MD (95% CI)	ES (d)
Step count	Baseline	5353 (3161)	n/a	n/a	5547 (3162)	n/a	n/a
	2 weeks	5654 (3440)	− 301 (− 1931 to 1330)	0.09	5905 (3249)	− 358 (− 1982 to 1265)	0.11
	4 weeks	5025 (3089)	328 (− 1315 to 1970)	0.1	5617 (3436)	− 70 (− 1746 to 1605)	0.02
	4 weeks later	6243 (3319)	− 891 (− 2527 to 745)	0.27	6146 (3432)	− 599 (− 2268 to 1069)	0.18
MET hours	Baseline	1.39 (0.15)	n/a	n/a	1.45 (0.14)	n/a	n/a
	2 weeks	1.35 (0.21)	0.05 (− 0.04 to 0.13)	0.21	1.41 (0.15)	0.04 (− 0.04 to 0.12)	0.28
	4 weeks	1.40 (0.18)	0.03 (− 0.09 to 0.08)	0.06	1.44 (0.17)	0.01 (− 0.07 to 0.09)	0.06
	4 weeks later	1.44 (0.17)	0.03 (− 0.13 to 0.04)	0.31	1.40 (0.20)	0.05 (− 0.03 to 0.13)	0.28

SD, standard deviation; MD, mean difference (baseline minus each time point); CI, confidence interval; ES, effect size; d, Cohen d; MET, metabolic equivalents.

*Statistically significant difference, *P* < 0.05.

### Visual analog scale (VAS) score for knee pain

The TENS and sham TENS groups reported decreased VAS scores during activity throughout the intervention. Specifically, the VAS scores in both groups were lower than the baseline values, with a particularly notable reduction observed in the TENS group. These observations were supported by the primary results of the two-way mixed model analysis of variance (ANOVA), which revealed interactions (F_3, 364.28_ = 6.41, *P* < 0.001) and a main effect of time (F_3, 364.28_ = 69.73, *P* < 0.001). No main effect of group was observed (F_1, 123.57_ = 3.11, *P* = 0.080).

Post-hoc tests revealed that in the TENS group, the VAS score was decreased at 2 weeks, 4 weeks, and 4 weeks later compared with the baseline values (*P* < 0.001). Additionally, the scores at 4 weeks and 4 weeks later were decreased compared with those at 2 weeks (*P* < 0.001 and *P* = 0.002, respectively). In the sham TENS group, the VAS score was decreased at 2 weeks, 4 weeks, and 4 weeks later compared with the baseline values (*P* = 0.009, *P* < 0.001, and *P* < 0.001, respectively). Moreover, the score reported 4 weeks later decreased compared with that reported at 2 weeks (*P* = 0.008).

A comparison of the groups at 2 and 4 weeks revealed that the VAS scores of the TENS group were lower than those of the sham TENS group (*P* = 0.048 and *P* < 0.001, respectively). Notably, no difference was reported 4 weeks later.

### Knee extensor strength

The TENS group showed increased peak force in the painful knee extensors. In contrast, no changes in force were observed in the non-painful knee. A two-way mixed model ANOVA revealed a significant interaction between groups and time for knee extensor strength in the painful knee (F_3, 363.91_ = 4.90, *P* = 0.020). A significant main effect of time was also observed (F_3, 363.91_ = 12.98, *P* < 0.001); however, no main effect of group was found (F_1, 123.76_ = 1.16, *P* = 0.283). Additionally, no significant interaction between groups and time was observed for knee extensor strength in the non-painful knee (F_3, 363.01_ = 1.68, *P* = 0.170).

Post-hoc tests revealed that the TENS group showed increased force in the painful knee at 4 weeks and 4 weeks later compared with the baseline values (*P* < 0.001). Additionally, the force recorded at 4 weeks was increased compared with that recorded at 2 weeks (*P* < 0.001). No significant results were obtained in the sham TENS group.

A comparison of the groups at 4 weeks revealed that the force recorded in the TENS group was higher than that in the sham TENS group (*P* = 0.034).

### Gait ability

No interactions were observed between groups and gait speed and timed up-and-go (TUG) test times (F_3, 364.65_ = 2.51, *P* = 0.058 and F_3, 365.19_ = 2.28, *P* = 0.079, respectively).

### Physical activity

Table 3 presents the results for each group along with the estimated effect sizes with their 95% confidence intervals. No interactions were observed between groups and time for step count or metabolic equivalents (MET) hours (step count: F_3, 338.38_ = 0.08, *P* = 0.972; MET hours: F_3, 328.58_ = 0.57, *P* = 0.638).

### Pain disability assessment scale (PDAS)

No interaction was observed between groups and time on the PDAS score (F_3, 363.27_ = 1.04, *P* = 0.374).

### Hospital anxiety and depression scale (HADS)

No interactions were seen between groups and time on each anxiety and depression score (F_3, 364.21_ = 1.02, *P* = 0.384 and F_3, 363.06_ = 0.74, *P* = 0.526, respectively).

### Pain catastrophizing scale (PCS)

No interactions were observed between groups and time on the PCS score (F_3, 363.87_ = 1.10, *P* = 0.351).

## Discussion

The findings of this study provide partial support for our hypotheses. Our study demonstrated that a 4-week intervention plan of TENS combined with home exercise significantly improved knee pain and extensor strength in community-dwelling older adults with pre-radiographic to mild knee osteoarthritis. However, our secondary hypothesis was not supported. The observed benefits were specific to pain and strength, as they did not translate to broader improvements in functional or psychosocial outcomes. These findings suggest that while combining TENS with home exercise is beneficial, its clinical impact is targeted; it does not provide a comprehensive multimodal benefit in this high-functioning population. The quality of our trial was ensured through strict adherence to the Consolidated Standards of Reporting Trials (CONSORT) statement, which bolstered the reliability and credibility of our findings^23^.

Recommendations of TENS for knee osteoarthritis vary across guidelines, ranging from conditional recommendations to strong caution against its use^6^. In a recent study, no additional benefits were observed when TENS was applied for treating participants with Kellgren–Lawrence grade ≥ 2 knee pain^20^. Based on this finding and our results, the combination of TENS and exercise therapy may be more effective in patients with pre-radiographic to mild osteoarthritis than in those with advanced knee osteoarthritis accompanied by bone deformity. This insight is crucial for developing targeted intervention strategies, as it highlights the potential for optimizing treatment efficacy based on the stage of osteoarthritis progression.

Previous research has shown that the minimal clinically important difference (MCID) for improving VAS pain scores is 2 out of 10 cm^24,25^. In this study, the TENS group exhibited differences that surpassed this MCID at all timepoints from 2 weeks to 4 weeks later when compared with baseline. Although the sham TENS group also demonstrated significant reductions in scores at all timepoints in comparison with baseline values, these changes did not reach the MCID. These results suggest that incorporating TENS into home exercise routines can be an effective strategy for managing knee pain and improving muscle strength in older adults.

We observed no significant changes in gait ability over time or between conditions. One possible reason for this finding is the baseline condition of the participants. For instance, a previous meta-analysis reported that the average gait speed of adults aged 65–80 years is 1.13–1.34 m/s^26^. However, despite having knee pain, the participants in the present study exhibited much faster average gait speeds of 1.84 m/s and 1.87 m/s in the TENS and sham TENS groups, respectively. This indicates that the cohort is highly functional and likely experienced ceiling effects. Similarly, previous studies have indicated that the TUG test results for community-dwelling older adults in the same age group range from 8 to 9 s^26^. However, the average TUG test times of both groups in the present study were in the 7-second range, further suggesting a ceiling effect.

Similar to the results regarding gait ability, the PDAS, HADS, and PCS scores did not change significantly. According to a previous study, a PDAS score of ≥ 10 points indicates confirmed anxiety or depression^27^. For the total HADS score, which is the sum of the depression and anxiety subscale scores, a score > 8 indicates the presence of depression^28^. Patients with a PCS score > 24 and < 15 are classified as pain catastrophizers^29^. In the present study, the baseline PDAS scores of the TENS and sham TENS groups were 9.39 and 9.65, respectively; however, the score decreased to 5.97 and 9.63, respectively, after 4 weeks. The baseline HADS score (sum of HADS-A and HADS-D scores) of the TENS group was 8.96, whereas that of the sham TENS group was 10.61. After the intervention, the HADS score of the TENS and sham TENS groups changed to 7.15 and 10.35, respectively. The baseline PCS scores of the TENS and sham TENS groups were 16.06 and 18.80, respectively. After the intervention, the PCS scores of the TENS and sham TENS groups changed to 14.70 and 19.43, respectively. These results suggest that, although participants exhibited chronic knee pain, as well as symptoms of depression and pain catastrophizing at baseline, the intervention might have had a minimal impact on pain intensity and emotional well-being. This limited effect may suggest that further strategies may be needed to more effectively target psychological and emotional factors in knee osteoarthritis management.

Several factors may explain why significant reduction in the VAS score did not translate to improved mental health outcomes in our study. First, interventions targeting psychological states, such as anxiety and depression, are consistently designed for longer durations. A previous review reported that psychotherapy is traditionally delivered by mental health professionals in weekly 60–90-minute sessions over a period of 8–12 weeks^30^. This suggests that our 4-week intervention period may have been insufficient to influence these more established psychological states. Second, our high-functioning cohort had low baseline psychological distress, which may have created a ceiling effect. A recent study reported MCIDs of 3.8 and 3.3 for the HADS-A and HADS-D subscales, respectively^31^. Our cohort’s baseline HADS-A and HADS-D scores were approximately 3.8 and 5.2, respectively. These low initial scores made it statistically and clinically difficult to detect meaningful improvement.

Previous reviews have shown that psychosocial interventions, such as self-management education and cognitive behavioral therapy, effectively reduce pain intensity, improve disability, enhance psychological outcomes, and boost the ability to perform activities of daily living in older adults with chronic pain^32–34^. Moreover, exercise along with psychosocial interventions reduces pain intensity and helps to maintain one’s ability to perform daily activities by enhancing psychological well-being and physical activity levels^32^. Therefore, it may be necessary to incorporate psychosocial interventions into the existing treatments for knee pain to further improve the psychological statuses of patients and their engagement in activities of daily living.

This study has some limitations. First, the scope of the study was limited to evaluating outcomes only up to 4 weeks post-intervention. Therefore, further research is needed to assess the long-term benefits of TENS for knee osteoarthritis. Moreover, the participants had pre-radiographic to mild osteoarthritis. Recruitment of participants through a public online advertisement likely introduced a substantial selection bias, resulting in a high-functioning cohort with unusually high baseline gait speeds. Together, these factors limit the generalizability of the findings to individuals with more severe knee osteoarthritis. Finally, the individualized nature of the home exercise protocol, while chosen for its clinical efficacy^34^, introduced heterogeneity in the specific exercises performed. This variability is a potential source of bias and may limit direct comparability with studies that use a strictly standardized protocol.

In conclusion, TENS combined with home exercise significantly improved knee pain and extensor strength in high-functioning older participants with pre-radiographic to mild knee osteoarthritis. However, as our participants were high-functioning, these findings may not be generalizable to frail or sedentary populations. Thus, future studies are warranted to evaluate whether similar benefits can be observed in more diverse cohorts of older adults with knee osteoarthritis.

## Methods

### Study design and participants

This was a randomized, double-masked, sham-controlled trial. Older participants experiencing knee pain were identified through an email survey and invited to visit the research facility at our university between January and June 2022. All recruited participants had a history of unilateral or bilateral knee pain. The eligibility criteria were individuals aged between 65 and 80 years with knee pain while walking (scored between 1 and 8 points on a 10-point scale) and symptomatic knee osteoarthritis classified as Kellgren–Lawrence grade ≤ 2. Individuals with the following conditions were excluded: (i) severe sensory impairment; (ii) dermatological conditions; (iii) conditions hindering exercise (e.g., central nervous system disorders, cardiovascular disease, internal medicine disorders, or orthopedic disorders); (iv) cognitive impairment (Mini-Mental State Examination score of ≤ 23); (v) contraindications for TENS, such as the presence of pacemakers or history of shunt surgery; (vi) history of psychiatric or neurological disorders; (vii) history of epileptic seizures; (viii) use of analgesics; and (ix) receipt of knee rehabilitation interventions before participation. The target sample size was calculated using G*power software^35^. Based on an effect size (d = 0.45) from a previous study on the immediate effects of the same TENS device used in this study^16^, a total of 85 participants were required to achieve 80% power at a significance level of 0.05. Accounting for a dropout rate of 10%, the final target sample size was calculated to be 95 participants.

This study was performed in accordance with the guidelines of the Declaration of Helsinki. It was approved by the Ethics Committee of our university, Tokyo, Japan (approval number: 20 − 014) and registered in the University Hospital Medical Information Network (registration number UMIN000043691; registered on March 22, 2021). Data collection was performed at the research laboratory of our university. Written informed consent was obtained from each participant before data collection began. This study was reported in accordance with the guidelines in the CONSORT statement^23^.

### Interventions

The intervention conditions were concealed from the participants and assessors. Participants were randomly assigned to a TENS or sham TENS group using float numbers between 0 and 1 from a continuous uniform distribution. Group assignment was based on whether the number drawn was ≤ 0.5 or > 0.5. The numbers were generated by a third party unrelated to the evaluation or intervention (Microsoft Excel, Redmond, WA, USA). The principal investigator, a physical therapist who was not involved in the assessment, conducted the enrollment, assignment, and randomization processes. The TENS group received TENS and performed home exercises, whereas the sham TENS group received sham TENS and performed home exercises. TENS was conducted for 2 h per day during, before, and after the home exercise therapy. The exercises were performed 5 days a week for 4 weeks.

### TENS

TENS was applied to the painful knee using a HV-F710 device (Omron Healthcare Co., Ltd., Kyoto, Japan). If the participant had bilateral knee pain, TENS was applied to the knee with more pain (Kellgren–Lawrence grade ≤ 2). The setting of the TENS therapy was based on its immediate effects on pain and physical performance^16^, which included a sweep mode ranging from 1 to 250 Hz, symmetrical biphasic pulse, and pulse width of 60 µs^16,36^. For participants in the TENS group, the intensity was adjusted gradually to reach a strong but comfortable tingling sensation just below the visible motor threshold, ensuring the stimulation was non-painful^16,36,37^. For participants in the sham TENS group, sham stimulation from an unconnected channel was applied with gradually increasing intensity while the intensity indicator illuminated as though the device was active. The participants were told they would not feel any current because the intended effects were below the sensory threshold. The TENS and the sham TENS devices were identical in appearance. Each device was packaged in a bag, labeled with a sequential number according to the randomization schedule, and provided to participants based on their assigned number. The allocation list was accessible only to the principal investigator, who was not involved in outcome assessment, until study completion, after which the data were locked.

### Home exercise therapy

The exercise interventions followed the methods described in a previous study^10^. The physical therapist instructed all participants on how to perform a home exercise program. The participants were trained to perform three exercises from a set of 10, based on an interview assessing their symptoms. The exercises included isotonic and isometric movements for the quadriceps, hip extension, abduction, adduction, squats, and stretches. Using the affected leg, participants performed their selected exercises for 20 min a day, 5 days a week, for 4 weeks. To minimize variability, while the selection of exercises was tailored to each participant, all exercises were chosen from a standardized list with uniform instructions on the technique, sets, and repetitions. Progression of the exercises followed a common, predefined protocol for all participants.

### Outcome measures

The primary outcome was the post-intervention changes in knee pain during activity, measured using the VAS. The secondary outcomes were the post-intervention changes in the knee extensor strength, gait ability, physical activities, and PDAS, HADS, and PCS scores. All assessments were conducted by a physical therapist at our university before the intervention (baseline), 2 weeks after the initiation of the intervention (2 weeks), 4 weeks after the initiation of the intervention (4 weeks), and 4 weeks after the end of the intervention (4 weeks later).

### Primary outcome

The causes of the most severe knee pain during activity were assessed through an interview conducted during the initial intervention, and the pain was measured using the VAS. Participants indicated their knee pain level by placing a vertical mark on a 10-cm horizontal line, where 0 cm represented “no pain” and 10 cm represented “the worst imaginable pain”^38,39^. The distance from the 0-cm point to the mark was then measured and recorded to the nearest tenth of a centimeter.

### Secondary outcomes

#### Knee extensor strength

Isometric knee extensor strength was assessed using a handheld dynamometer (HHD) with a pull sensor (MT-100; SAKAImed Co., Ltd., Tokyo, Japan)^16^. The maximum force, recorded in Newtons, was measured twice for each participant. Testing began with participants seated on a therapy bed with their knees at 90° flexion. The HHD was positioned proximal to the lateral malleolus and secured with an inelastic strap fastened around the bed. The participants were instructed to extend their legs for 5 s, and strong verbal encouragement was provided to ensure maximum effort.

#### Gait ability

The 10-m walk and TUG tests were used to measure gait ability. In the 10-m walk test, the participants were instructed to walk 10 m as fast as possible while feeling safe, ensuring they maintained their natural gait pattern and avoided running. In the TUG test, participants were asked to stand up from a chair, walk 3 m in a straight line as quickly as possible, circle a cone on the floor, and return to sit in the chair^40^.

#### Physical activities

All participants were instructed to wear an activity monitor (HJA-750 C; Omron Healthcare Co., Ltd.) on their lower back from waking until bedtime (excluding periods of bathing) over 4 weeks before the intervention and an additional 4 weeks afterward to track their daily physical activity. The monitor recorded data on step count, activity intensity, and duration of wear^38^. To calculate weekly medians, the measurement period was divided into weekly intervals starting from the onset of monitoring. The activity intensity was defined based on METs. MET hours were calculated by multiplying each MET value by the duration of each activity, normalized by the total wear time.

#### PDAS

The PDAS evaluates the extent of pain interference in daily activities over the past week^27,41^. The scale comprises 20 items, each rated on a Likert scale from 0 (“pain did not interfere with this activity”) to 4 (“pain interfered completely with this activity”). The total score ranges from 0 to 60, with higher scores indicating greater levels of pain interference.

#### HADS

Participants were required to complete the questionnaire, which comprised 14 questions (7 for anxiety and 7 for depression), each rated on a Likert scale from 0 to 3. The scores for the subscales were summed and analyzed separately. Total scores range from 0 to 21, and higher scores indicate greater anxiety or depression levels^28,42,43^.

#### PCS

The PCS comprises 13 items and three subscales^29^. To use this scale, participants are required to rate the degree to which they have catastrophizing thoughts and feelings when experiencing pain on a 5-point Likert scale. The total score is computed by summing the points for the response to each item. Total scores range from 0 to 52, with higher scores representing greater catastrophic thinking in response to pain^44^.

### Statistical analysis

Analyses were performed in accordance with the intention-to-treat principle. We used a linear mixed model based on two-way ANOVA to evaluate the differences in outcomes (VAS score, gait ability, knee extensor strength, step count, MET hours, and PDAS, HADS, and PCS scores) between the groups (TENS and sham TENS) and time points (baseline, 2 weeks, 4 weeks, and 4 weeks later). All participants who were randomly assigned were included. The participants were included as random effects in all mixed models. When ANOVA showed significant main effects and interactions, further investigations were performed using t-tests with Bonferroni adjustments for multiple comparisons. The chi-square test was used to assess the success of participant masking. Participants with missing data for this question were excluded from the analysis that evaluated the association between the participants’ actual group assignment (TENS or sham TENS group) and their guessed group assignment (TENS or sham TENS group). For this analysis, participants who answered, “Don’t Know” on the questionnaire were treated as having guessed incorrectly. *P*-values of < 0.05 were considered statistically significant. All analyses were performed using SPSS 29.0 (IBM Corp., New York, NY, USA) for Windows.

## Data Availability

The datasets used and/or analyzed during the current study are available from the corresponding author on reasonable request.
